# Supplementary material for: Study on the allocation efficiency of medical and health resources in Hainan Province: Based on the super-efficiency SBM—Malmquist model
Source: PLoS One. 2024 Feb 29;19(2):e0294774. doi: 10.1371/journal.pone.0294774 (PMC10903814; doi:10.1371/journal.pone.0294774)
Supplement: Attachment — Submitted filename: Response to Reviewers.docx [file pone.0294774.s001.docx]

**Dear Reviewers:**

Thank you very much for your comments and professional advice on the manuscript (Title: Study on the allocation efficiency of medical and health resources in Hainan Province under the construction of Free Trade Port: Based on the super-efficiency SBM — Malmquist model. Number: PONE-D-23-10563). These opinions are helpful to improve the academic and rigorous nature of the manuscript. In this regard, we hold a serious and steadfast learning attitude. According to your suggestions, we have carefully revised the manuscript and adjusted and polished some of the language. We hope the manuscript can be better. We have not changed much in the overall framework, but we have changed the "Conclusion and policy implications" section to "Conclusions and suggestions" because the term is more consistent with our manuscript. In addition, the "Introduction" section, the "Discussion" section, the "Conclusions and suggestions" section and the "limitations" section have also been greatly revised, which are the focus of attention of experts. In a marked revision manuscript, we have highlighted your proposed revision in yellow and the problems we found and the appropriate changes to the original text in red. Also, in this reply, we have highlighted some of the content in gray so that you can look more clear. Specific modification details are as follows:

**Reviewer #1:**

Thank you very much for your recognition of the manuscript and your professional suggestions. The following are our amendments and replies.

1. The manuscript titled "Equity and Efficiency of Medical and Health Resource Allocation: A Case Study of Hainan Province" provides valuable insights into the allocation of medical and health resources in Hainan Province, China. The authors effectively address the significance of equity and efficiency in resource allocation, highlighting the impact on sustainable and high-quality development of health services. The study aims to improve resource allocation efficiency and provide references for other regions. This manuscript contributes to the literature on health resource allocation and offers meaningful recommendations for optimizing resource utilization in Hainan Province. Overall, the manuscript is well-written and presents a systematic analysis of medical and health resource allocation in Hainan Province. However, it is important to note that the manuscript could benefit from further discussion on the limitations of the study, particularly regarding the influence of environmental factors on resource allocation and the missing data in some regions. Addressing these limitations would enhance the robustness of the research.

**The author's answer：**Thank you very much for your recognition of the manuscript and your professional suggestions. Based on your suggestions, we have had a more specific discussion about the limitations. (Lines 371-384) The specific changes are as follows:

**Original text：**However, there are some limitations in this study, which do not further investigate the influence of environmental factors on the input and output index. There are some missing data in individual regions, which cannot be fully covered. It is hoped to improve it in the later period.

**The modified：**However, this study has some limitations. First, the impact of environmental factors on input-output indicators is not further explored. This may lead to a discrepancy between the estimated results and the actual situation. Factors such as economy, population, policy, health personnel, geographical location, transport and education can have a significant impact on the efficiency of health resource allocation. Regions with relatively good environmental factors will have more policy support, greater demand for health care and more economic and financial input. This results in a relatively high efficiency of healthcare resource allocation. Secondly, there are some missing data in individual regions that cannot be fully covered. This may result in not reflecting the overall situation of health resource allocation efficiency in Hainan Province, making the sample incomplete or biased, which may cause errors in the conclusions and affect the robustness of the results. The focus of the later project is to study the influencing factors and the degree of influence on the allocation efficiency of health resources in Hainan Province, while the missing data will be made up by in-depth research or sensitivity analysis, etc. to ensure that the data are more robust.

2. How did the study measure the comprehensive efficiency of medical and health resource allocation in Hainan Province?

**The author's answer：**We are sorry that we only wrote about the measure of dynamic efficiency and total factor productivity in "Data and methods" in the paper, but did not explicitly write how to measure comprehensive efficiency. Thank you for spotting this. We have added this part to the "Super-efficiency SBM" section of the text. (Lines 168-172) Specifically add as follows:

**Added content:** According to the super-efficiency SBM model, ρ < 1 indicates that the comprehensive efficiency of resource allocation is in a relatively invalid state and the efficiency is low. ρ ≥ 1 indicates that the comprehensive efficiency of resource allocation is in a relatively effective state and the efficiency is high. It is generally believed that the larger the comprehensive efficiency value, the higher the efficiency of resource allocation, and the better the level.

3. What specific factors contribute to the decline in the allocation efficiency of medical and health resources in Hainan Province, particularly during the years 2019 and 2020?

**The author's answer：**The factors leading to the decline in the efficiency of medical and health resource allocation in Hainan Province include policy, economic development, population, geography and transportation. The reasons are also mentioned in the "Discussion" section of this manuscript (lines 255-278), but may need to be more specific. Therefore, we have made a more detailed supplement to the first paragraph of "Discussion" for readers to understand. Mainly, the second and third reasons are supplemented in detail. Specific modifications are as follows:

**Original text：**First, in 2017, it was emphasized in the Key Tasks of Deepening the Reform of the Medical and Health System in 2017 that the year 2017 was a key year for China to form a systematic framework of the basic medical and health system and complete the phased objectives of the medical reform. Therefore, China promulgated a number of medical reform policies, such as the cancellation of the drug price addition policy, the comprehensive launch of the medical consortium pilot work, and the implementation of a hierarchical diagnosis and treatment system. The medical and health service system in our country has broken the inherent mode under the effects of this series of medical reforms. During the initial period of the implementation of the new and old policies, it has exerted influence on the normal operation of health undertakings. Second, it may have something to do with the construction of free trade ports in Hainan Province starting in 2018. Hainan has given priority to institutional integration and innovation in its development. Various preferential policies have been formulated and introduced to attract capital, talent, high-end equipment, and other resources to Hainan, breaking the state of self-sufficiency in the past. The reform of the medical system has been deepened, and the medical and health resources are being restructured. Third, the impact of the global COVID-19 pandemic has led to low efficiency of medical resources.

**The modified：**First, in 2017, it was emphasized in the Key Tasks of Deepening the Reform of the Medical and Health System in 2017 that the year 2017 was a key year for China to form a systematic framework of the basic medical and health system and complete the phased objectives of the medical reform. Therefore, China promulgated a number of medical reform policies, such as the cancellation of the drug price addition policy, the comprehensive launch of the medical consortium pilot work, and the implementation of a hierarchical diagnosis and treatment system. The medical and health service system in our country has broken the inherent mode under the effects of this series of medical reforms. During the initial period of the implementation of the new and old policies, it has exerted influence on the normal operation of health undertakings. Second, it may have something to do with the construction of free trade ports in Hainan Province starting in 2018. Hainan has given priority to institutional integration and innovation in its development. Various preferential policies have been formulated and introduced to attract capital, talent, high-end equipment, and other resources to Hainan, breaking the state of self-sufficiency in the past. With the deepening of the reform of the medical system, medical and health resources are being restructured and adjusted, and there is a phenomenon of underutilization and reasonable operation, resulting in a decline in efficiency instead of improvement. This also reflects from another side that no matter how much health resources are invested, if the resources are not reasonably and fully utilized, the efficiency of resource allocation will decline and the output will be poor. Third, due to the impact of the global COVID-19 epidemic in 2019–2020, health resources have been urgently deployed, including equipment, beds, personnel and other resources, focusing on the allocation of the most affected areas. More beds were transferred to treat COVID-19 patients. Medical resources are diverted to emergency response to the epidemic, thus affecting the original efficiency.

4. Are there any specific policies or strategies mentioned in the manuscript that address the issues identified in the allocation efficiency of medical and health resources in Hainan Province?

**The author's answer：**Yes, in view of the problems existing in the efficiency of medical and health resource allocation in Hainan Province, it is important to reflect in the "14th Five-Year Plan for Hygiene and Health of Hainan Province", "14th Five-Year Plan for Grassroots Hygiene and Health of Hainan Province", "14th Five-Year Plan for Digital Health Development of Hainan Province" and other policies. The article lists specific strategies to improve efficiency in the "Conclusions and Suggestions" part. But that may not be specific enough. Based on the actual situation in Hainan Province, we modified the strategy in more detail to make it more operable (lines 313-359). The basic framework and keywords have not changed more, but the relevant content has been expanded to be more specific. It mainly includes the system innovation, the balance of medical and health resources, the cultivation and introduction of health talents, and the exchange and cooperation at home and abroad. The details are as follows:

**Original text：**First, Hainan should continue to take institutional integration and innovation as the "starting point" to improve the ability of modern governance of health services. Hainan Province should accelerate the implementation and optimization of policies, accelerate the progress of medical reform, the development of Boao International Medical Tourism Pilot Zone and the development of the healthcare industry, attach importance to medical innovation and achievement transformation, and promote the health industry to enter the fast track of high-quality development steadily. Second, Hainan should balance the distribution and optimization of health resources, and speed up the process of promoting graded diagnosis and treatment and "Internet Plus" medical treatment. Strengthen supervision and management, appropriately expand the scale of medical services in big cities, and for inefficient cities and counties, and improve the efficiency of service utilization by means of technology introduction, graded diagnosis and treatment, and "Internet +" medical treatment while ensuring adequate input. To speed up the development of Haikou, Sanya, and Danyang economic circles, to form a new pattern of development, and to improve medical consortium, hospital groups, and other models of division of labor and collaboration. Third, strengthen cooperation and exchanges and continue to upgrade hardware elements. To maintain close contact with Singapore, Shanghai, Beijing, Guangdong Province, and other places and strengthen technical exchanges, and improve the level of medical technology and service efficiency. First-class medical institutions and research centers at home and abroad are introduced to Hainan to achieve joint construction, governance, and sharing, and services for the construction of the Hainan Free trade Port. Fourth, accelerate the introduction and training of health professionals to enhance the soft power of health. On the one hand, in order to adapt to the development of the Hainan Free Trade Port, it needs to increase the total number of health professionals. On the other hand, relying on favorable policies, Hainan will focus on introducing and training high-level health professionals, mastering the core medical technology, and creating high-end medical services in the world. In addition, the policy should be appropriately tilted to the community level to speed up the construction of health professionals at the community level and guarantee their treatment. Improving the allocation efficiency of medical and health resources is an important guarantee for promoting the development of the health industry of the Hainan Free Trade Port, and an inevitable requirement for the high-quality construction of the Hainan Free Trade Port.

**The modified：**First, Hainan should continue to take institutional integration and innovation as the "starting point" to improve the ability of modern governance of health services. The "14th Five-Year Plan for Hygiene and Health of Hainan Province" issued by Hainan Province in 2021 emphasizes that Hainan must accelerate the expansion and balanced distribution of high-quality medical and health resources and improve the allocation efficiency of medical and health resources. The Hainan provincial government should further implement and optimize policies, accelerate the process of medical reform, improve detailed medical and health resource planning, and rationally allocate medical and health hardware and software resources according to population, geography, demand, and other factors in order to improve efficiency. Accelerate the development of key health projects in Hainan Province. For example, promote the implementation of the global "One Health" demonstration project in Hainan Province through publicity and improvement of the medical and prevention integration governance model; Through the high-level equipment of hardware and software facilities, increase the degree of policy openness, pay attention to medical innovation and achievement transformation, and promote the process of the Boao Lecheng International Medical Tourism Pilot Zone; Based on the unique natural environment of Hainan Province, it is necessary to promote the rational development of the healthcare industry.

Second, Hainan should balance the layout and optimize the allocation of health resources, and speed up the process of promoting graded diagnosis and treatment and "Internet Plus" medical treatment. Strengthen supervision and management, appropriately expand the scale of medical services in big cities, and for inefficient cities and counties, and improve the efficiency of service utilization by means of technology introduction, graded diagnosis and treatment, and "Internet +" medical treatment while ensuring adequate input. Speed up the building of economic circles in Haikou, Sanya, and Dan Ocean, form a point with a circle and a circle with an area, promote the formation of a new development pattern, accelerate the implementation of the relevant implementation opinions of Hainan Province's grid-tight medical and health service system, improve the medical consortium, hospital group, and other division of labor and cooperation models, and comprehensively improve diagnosis and treatment efficiency and resource utilization efficiency.

Third, accelerate the introduction and training of health professionals, expand the total number, and improve the quality. On the one hand, the establishment of first-class medical colleges, especially nursing colleges. Independently cultivate and stabilize health professionals. On the other hand, Hainan Province can rely on favorable policies to improve the salary of health professionals, innovate the post and staffing system, focus on introducing and training high-level health professionals, master the core medical technology, improve the level of medical technology, and create high-end medical services in the world. In addition, the policy should be appropriately tilted to the grass-roots level, accelerate the construction of the grass-roots health personnel team, innovate the assessment system of grass-roots health personnel, pay more attention to the improvement of their post-competence, service attitude, diagnosis and treatment level, and ensure the quality and quantity of grass-roots health personnel.

Fourth, strengthen international cooperation and exchanges to promote the sharing of medical and health resources. To maintain close contact with Beijing, Shanghai, Guangdong Province, and other places to strengthen technical exchanges and improve the level of medical technology and service efficiency. The introduction of domestic and foreign first-class medical institutions and research centers in Hainan to achieve co-construction, co-governance, and sharing to serve the construction of the Hainan Free Trade Port.

5. What evidence or examples are provided to support the claim that economically developed areas with large populations have relatively higher allocation efficiency of health resources in Hainan Province?

**The author's answer：**Haikou City is a relatively developed area in Hainan Province. Haikou City is the capital city of Hainan Province, and its annual GDP and population are the largest. Table 2 also shows that the comprehensive efficiency of Haikou City is the highest. To solve this problem, we can find some reasons in the following:

(1) High investment. Economically developed areas usually have a higher level of investment in health resources, including investment in medical facilities, equipment and personnel, which can provide better allocation and utilization of health resources and improve efficiency.
(2) Advanced technology and management experience. Economically developed regions usually have more advanced medical technology and management experience, which helps optimize the allocation of medical resources and improve efficiency. For example, the application of 5G technology and electronic medical records can promote hierarchical diagnosis and treatment and improve the efficiency of healthcare institutions. The research and development of new drugs and the improvement of surgical techniques can improve the efficiency of health resource allocation.
(3) Policy support. Developed regions usually have better health policies and can get greater policy preferences. For example, Haikou City has many targeted innovative measures of health system integration. Many of the pilot measures will be implemented first in Haikou.
(4) Patient needs. Areas with large populations usually have a higher demand for health services, which encourages governments and medical institutions to increase health investment. In addition, economically developed areas with large populations are likely to be better educated and more health-conscious, which will also promote efficient use of health resources.

**Reviewer #2:**

Thank you very much for your recognition of the manuscript and your professional suggestions. The following are our amendments and replies.

1. In the description of the province, the number of population should be given.

**The author's answer：**Thank you very much for your reminding. Yes, we quite agree with your suggestion. And we have to make changes. After consideration, we added the population of Hainan in 2020 to the description of the province in the "Introduction" section. (Line 104)

**Add content:**and the number of permanent residents in 2020 is 10,123,400 people.

2. The authors focus on human resources, and is it not worth considering other resources, there is also a budget constraint that affects the efficiency of management, and thus also clinical effectiveness.

**The author's answer：**Medical and health human resources are very important, they are the core and the key factor of health development. Medical and health human resources have an important impact on the development of health undertakings in Hainan Free Trade Port. Specific aspects may be as follows:

(1) Access to health services. Adequate human resources for health can provide a wider range of high-quality health services and ensure that the population has timely access to medical and health services. The development of Hainan Free Trade Port means the possibility of population flow and growth, so sufficient health human resources are needed to meet the growing demand for health services.
(2) Health status of the population. Whether the health human resources are sufficient or not will directly affect the health status of residents in Hainan Free Trade Port. Adequate doctors, nurses and other medical professionals can provide comprehensive medical services, prevent and control diseases, and improve the health of residents.
(3) Development of medical tourism. The Hainan Free Trade Port has developed medical tourism to attract patients from home and abroad to seek medical services in Hainan. This requires Hainan to have sufficient health human resources to provide high-level medical services and attract patients to seek medical treatment.
(4) Health security and epidemic prevention and control: Hainan Free Trade Port needs to have stronger health human resources to ensure health security and epidemic prevention and control. To ensure the health and safety of residents and visitors.

In short, health human resources have an important impact on the development of Hainan Free Trade Port, and are of great significance for providing high-quality health services, promoting the development of medical tourism, and ensuring health safety. Therefore, Hainan needs to pay attention to the training and allocation of health human resources to support the rapid development of free trade port.

But this does not mean focusing only on the health workforce, other resources are also very important. When selecting indicators in this study, there are also indicators related to equipment and facilities, (Lines 134-135) and the influence of personnel, capital and material will also be mentioned in the "Discussion" sections.(Lines 266-268) However, it is undeniable that this study does involve less capital (budget) input. In the index selection, capital input was not selected as one of the input indicators. This is because, considering that financial resources are almost transformed into human and material resources, if financial resources are added as input indicators, duplication may occur. And data on capital are harder to come by. There is no denying that capital is indeed a very important influencing factor, and we will focus on this factor in the later study of influencing factors.

3. It is incomprehensible to refer to a free port, what impact does it have on the functioning of health care? Are there any dependencies?

**The author's answer：**Hainan Province is currently the only free trade port with Chinese characteristics in mainland China. Under the background of free trade port, Hainan Province will have more development opportunities. The Hainan Free Trade Port may have the following impacts on the operation of health care:

(1) Provide more medical resources. The free trade port policy is expected to attract more domestic and foreign medical institutions and professionals to enter Hainan, increase the supply of medical resources, and improve the level and quality of medical services.

(2) Promote medical technology innovation. The free trade port policy encourages scientific and technological innovation and intellectual property protection, which helps attract medical technology and pharmaceutical innovation enterprises to enter Hainan and promote the development and application of medical technology.

(3) Improve the quality of medical services. The construction of the free trade port will promote the competition between medical institutions and medical personnel, forcing them to improve the quality of medical services and provide better medical experience to patients.

(4) Promote the development of medical tourism. The free trade port policy is expected to attract more international patients to Hainan for medical treatment, promote the development of medical tourism, and increase the demand for medical services and economic income in Hainan.

(5) Optimize the medical management system: The construction of the free trade port may bring about the reform and innovation of the medical management system, promote the standardization and modernization of medical institutions, and improve the operation efficiency and management level.

4. Introducing solutions that work well in health care in one country does not lead to success in another country. Therefore, it is incomprehensible to refer to Singapore. The differences between the Chinese province and Singapore are so large that the solutions from Singapore cannot be expected to have positive effects in China. Thorough research is definitely needed.

**The author's answer：**Thank you very much for your advice. We agree with your statement. Every region is different. This article mentions Singapore in both the "Introduction" and "Conclusions and suggestions" sections. After careful review and consideration, we have revised the article. In the "Introduction", Singapore's experience is mainly derived from a piece of literature, and it may be more suitable for this paper to put this literature into domestic and foreign research, rather than taking Singapore out alone. (Lines 79-82) In the section of "Conclusions and suggestions", the reference to Singapore's experience is removed. (Line 355-356) It is not because Singapore has no experience to learn from, but we agree with your opinion. At the same time, we consider that most of Singapore's medical institutions are private, and there is a big difference between Singapore and China. Therefore, the experience of other excellent regions in China is more in line with the development trend of Hainan Province.

The world is open and inclusive in many ways. Singapore has been relatively successful in the process of building a free trade port. Singapore has done better in primary health care and community services. If we have the opportunity, we will thoroughly study its specific practices and see if it can bring some inspiration to the construction of Hainan Free Trade Port. We really appreciate your advice.

**Original text：**In Sayem Ahmed's study on the efficiency of health systems in Asia, Singapore's efficiency is effective, indicating that it makes full use of health system resources.[4] Singapore is also a free trade port, which is worth learning from Hainan Free Trade Port.

**The modified：**Sayem Ahmed ( 2019 ) [4] used the DEA, Tobit model, and smooth bootstrap model to evaluate the technical efficiency of health systems in Asian countries and found that up to 91.3% of countries had inefficient utilization of health resources. (Lines 79-82)

**Original text：**strengthen cooperation and exchanges and continue to upgrade hardware elements. To maintain close contact with Singapore, Shanghai, Beijing, Guangdong Province, and other places and strengthen technical exchanges,

**The modified：**To maintain close contact with Beijing, Shanghai, Guangdong Province, and other places to strengthen technical exchanges and improve the level of medical technology and service efficiency. (Line 355-356)

5. The introduction should include a more detailed description of the study itself.

**The author's answer：**Thank you very much for your advice. It is true that the "Introduction" part of this study is not good enough. We revised it in detail to make it more specific and clear. In the "Introduction" part, we focus on the research theme, writing the research background, significance, domestic and foreign research, methodological research and research purpose. (Lines 46-123) The specific changes are as follows:

**Original text：**The contradiction between the finiteness of health resources and the infinite demand of people is prominent.[1] How to make more effective use of the limited health resources, meet the needs of people at different levels of health services, optimize the distribution of health resources and boost the efficiency of use is a common problem faced by the global medical and health reform.[1, 2] According to the "Healthy China 2030" Plan's Outline in 2016, by 2030, China's health institutions and systems will be improved, the development of the health sector will be more coordinated, healthy lifestyles will be popularized, and the quality and guarantee of health services will continue to improve. The important premise of improving health levels and promoting health equity is the rational allocation and efficient utilization of medical and health resources. Hainan Province is the only free trade port in China and is also known as a "test bed for national reform and opening up ", which means that the successful experience of Hainan's reforms may be extended to other parts of China, and its construction is of great significance to China.[3] In Sayem Ahmed's study on the efficiency of health systems in Asia, Singapore's efficiency is effective, indicating that it makes full use of health system resources.[4] Singapore is also a free trade port, which is worth learning from Hainan Free Trade Port.

Hainan Province (108°37 '-- 111°03' east longitude, 18°10 '-- 20°10' north latitude) is located in the southernmost part of China, close to Southeast Asia, with a total land area of 35,400 square kilometers. In 2018, the Party Central Committee decided to support Hainan in gradually exploring and steadily deepening the construction of a free trade port with Chinese characteristics, which has helped the province comprehensively deepen reform. Hainan is committed to building Health Island and Longevity Island. Under the free trade port policy, Hainan has focused on institutional integration innovation and made major breakthroughs in health care. However, with the increase in population, the intensification of aging, and the change of disease spectrum, the current medical and health resources in Hainan Province are obviously in shortage, and there are outstanding problems such as imbalance, inequity, low efficiency, and lack of human resources. In order to effectively cope with future changes, Hainan must speed up the adjustment and optimization of medical and health resources and improve the efficiency of resource utilization, so as to provide an important guarantee for the high-quality development of health services in Hainan and escort the construction of free trade ports. At present, more attention is paid to the ecological environment and trade of Hainan Province at home and abroad, while there are few related types of research on health status, especially those on Hainan Province under the policy of free trade port with Chinese characteristics. In this study, the super-efficiency SBM and Malmquist index model were used to study the allocation efficiency of medical and health resources in Hainan Province during 2016-2020, analyze the current situation, problems, and causes, and put forward reasonable suggestions to provide a reference for scientific health development planning.

**The modified：**The contradiction between the finiteness of health resources and the infinite demand of people is prominent.[1] How to make more effective use of the limited health resources, meet the needs of people at different levels of health services, optimize the distribution of health resources and boost the efficiency of use is a common problem faced by the global medical and health reform.[1, 2] According to the "Healthy China 2030" Plan's Outline in 2016, by 2030, China's health institutions and systems will be improved, the development of the health sector will be more coordinated, healthy lifestyles will be popularized, and the quality and guarantee of health services will continue to improve. The important premise of improving health levels and promoting health equity is the rational allocation and efficient utilization of medical and health resources. The improvement of the efficiency of the allocation of medical and health resources can not only promote the accessibility and quality of medical services but also play an important role in improving people's living standards and promoting economic growth. Since the implementation of China's new medical reform in 2009, the amount of health resource investment has generally increased, and great results have been achieved.

Foreign studies on the allocation of health resources started earlier, and the UK was the first country to conduct research on the allocation of health resources [3]. In 1976, the Resource Allocation Working Party (RAWP) was established in the United Kingdom to study the allocation of health resources. Research on the allocation of health resources in China began in the 1980s. With the development of the economy, the rapid increase in population, and the intensification of aging, people have put forward higher requirements for medical and health resources and services, and the government has continuously increased its investment in health resources. The number of health institutions, beds, and health technicians is increasing, and the service capacity of medical and health institutions is greatly improved, but the efficiency of medical and health resource allocation is still low, which may lead to increased investment in subsequent health resources but poor output. This not only fails to meet people's growing medical and health needs but also causes serious waste of medical and health resources, resulting in the rapid growth of medical costs, increasing people's medical burden, and restricting the sustainable and high-quality development of health and health undertakings, which is contrary to the ultimate goal of national health and health development.

How to make the best use of health resources is a hot topic for scholars at home and abroad. Data envelopment analysis ( DEA ) is the most mature and extensive method to study the efficiency of the allocation of medical and health resources. For example, Sayem Ahmed (2019) [4] used the DEA, Tobit model, and smooth bootstrap model to evaluate the technical efficiency of health systems in Asian countries and found that up to 91.3% of countries had inefficient utilization of health resources. Lacko R. (2022) [5] uses the DEA model and Malmquist index to compare the efficiency and productivity of EU countries. It is found that although countries joining the EU get a high technical efficiency value, the overall technical efficiency shows a downward trend, and the productivity of Western countries shows stagnation. Xiang Yingru (2020) [6] analyzed the efficiency of provincial health resources in China by using the BCC model of DEA and the Malmquist index. The study pointed out that after the implementation of medical reform in China, resources are still wasted, output is insufficient in some areas, and efficiency is low due to the excessive number of public hospitals and the serious aging of the population. Technological progress is a key factor affecting efficiency. Luo Liangqing (2008) [7] used the DEA model to study the production efficiency of medical and health services in the eastern, central, and western regions of China and found that the production efficiency of medical and health services in China was generally low. It can be seen from many related studies that the traditional DEA model has some limitations when studying the efficiency of the allocation of medical and health resources. Therefore, the method is improved in this article.

The problem of uneven development among different regions in China has always existed. Hainan Province is currently the only free trade port with Chinese characteristics on the Chinese mainland, and is also known as a "test bed for national reform and opening up ", which means that the successful experience of Hainan's reforms may be extended to other parts of China, and its construction is of great significance to China.[8] Hainan Province (108°37 '-- 111°03' east longitude, 18°10 '-- 20°10' north latitude) is located in the southernmost part of China, close to Southeast Asia, with a total land area of 35,400 square kilometers, and the number of permanent residents in 2020 is 10,123,400 people. In 2018, the Party Central Committee decided to support Hainan in gradually exploring and steadily deepening the construction of a free trade port with Chinese characteristics, which has helped the province comprehensively deepen reform. Hainan is committed to building Health Island and Longevity Island. Under the free trade port policy, Hainan has focused on institutional integration innovation and made major breakthroughs in health care. However, with the increase in population, the intensification of aging, and the change of disease spectrum, the current medical and health resources in Hainan Province are obviously in shortage, and there are outstanding problems such as imbalance, inequity, low efficiency, and lack of human resources. In order to effectively cope with future changes, Hainan must speed up the adjustment and optimization of medical and health resources and improve the efficiency of resource utilization, so as to provide an important guarantee for the high-quality development of health services in Hainan and escort the construction of free trade ports. At present, more attention is paid to the ecological environment and trade of Hainan Province at home and abroad, while there are few related types of research on health status, especially those on Hainan Province under the policy of free trade port with Chinese characteristics. In this study, the super-efficiency SBM model and Malmquist index were used to study the allocation efficiency of medical and health resources in Hainan Province during 2016-2020, analyze the current situation, problems, and causes, and put forward reasonable suggestions to provide a reference for scientific health development planning.

6. Institutions appear as input indicators. What institutions are we talking about? Are they supposed to be service providers?

**The author's answer：**The indexes selected by data envelopment analysis should be representative, deterministic, independent and stable. This study selects indicators on the basis of literature review. As an input indicator, medical and health institutions mainly include hospitals, primary medical and health institutions, professional public health institutions, internet hospitals, and other medical and health institutions in the Hainan Provincial Health Statistics Yearbook. It reflects the overall size of the region's health systems. It is a public place to provide medical and health services and is one of the carriers.

7. Policy conclusions are vaguely worded.

**The author's answer：**Thank you very much for your advice. We have reorganized the "Conclusions and suggestions" section with a view to making the recommendations more specific and actionable. (Lines 313–359) The basic framework and keywords have not changed much, but the relevant content has been expanded to be more specific. It mainly includes the system innovation, the balance of medical and health resources, the cultivation and introduction of health talents, and the exchange and cooperation at home and abroad. The specific changes are as follows:

**Original text：**First, Hainan should continue to take institutional integration and innovation as the "starting point" to improve the ability of modern governance of health services. Hainan Province should accelerate the implementation and optimization of policies, accelerate the progress of medical reform, the development of Boao International Medical Tourism Pilot Zone and the development of the healthcare industry, attach importance to medical innovation and achievement transformation, and promote the health industry to enter the fast track of high-quality development steadily. Second, Hainan should balance the distribution and optimization of health resources, and speed up the process of promoting graded diagnosis and treatment and "Internet Plus" medical treatment. Strengthen supervision and management, appropriately expand the scale of medical services in big cities, and for inefficient cities and counties, and improve the efficiency of service utilization by means of technology introduction, graded diagnosis and treatment, and "Internet +" medical treatment while ensuring adequate input. To speed up the development of Haikou, Sanya, and Danyang economic circles, to form a new pattern of development, and to improve medical consortium, hospital groups, and other models of division of labor and collaboration. Third, strengthen cooperation and exchanges and continue to upgrade hardware elements. To maintain close contact with Singapore, Shanghai, Beijing, Guangdong Province, and other places and strengthen technical exchanges, and improve the level of medical technology and service efficiency. First-class medical institutions and research centers at home and abroad are introduced to Hainan to achieve joint construction, governance, and sharing, and services for the construction of the Hainan Free trade Port. Fourth, accelerate the introduction and training of health professionals to enhance the soft power of health. On the one hand, in order to adapt to the development of the Hainan Free Trade Port, it needs to increase the total number of health professionals. On the other hand, relying on favorable policies, Hainan will focus on introducing and training high-level health professionals, mastering the core medical technology, and creating high-end medical services in the world. In addition, the policy should be appropriately tilted to the community level to speed up the construction of health professionals at the community level and guarantee their treatment. Improving the allocation efficiency of medical and health resources is an important guarantee for promoting the development of the health industry of the Hainan Free Trade Port, and an inevitable requirement for the high-quality construction of the Hainan Free Trade Port.

**The modified：**First, Hainan should continue to take institutional integration and innovation as the "starting point" to improve the ability of modern governance of health services. The "14th Five-Year Plan for Hygiene and Health of Hainan Province" issued by Hainan Province in 2021 emphasizes that Hainan must accelerate the expansion and balanced distribution of high-quality medical and health resources and improve the allocation efficiency of medical and health resources. The Hainan provincial government should further implement and optimize policies, accelerate the process of medical reform, improve detailed medical and health resource planning, and rationally allocate medical and health hardware and software resources according to population, geography, demand, and other factors in order to improve efficiency. Accelerate the development of key health projects in Hainan Province. For example, promote the implementation of the global "One Health" demonstration project in Hainan Province through publicity and improvement of the medical and prevention integration governance model; Through the high-level equipment of hardware and software facilities, increase the degree of policy openness, pay attention to medical innovation and achievement transformation, and promote the process of the Boao Lecheng International Medical Tourism Pilot Zone; Based on the unique natural environment of Hainan Province, it is necessary to promote the rational development of the healthcare industry.

Second, Hainan should balance the layout and optimize the allocation of health resources, and speed up the process of promoting graded diagnosis and treatment and "Internet Plus" medical treatment. Strengthen supervision and management, appropriately expand the scale of medical services in big cities, and for inefficient cities and counties, and improve the efficiency of service utilization by means of technology introduction, graded diagnosis and treatment, and "Internet +" medical treatment while ensuring adequate input. Speed up the building of economic circles in Haikou, Sanya, and Dan Ocean, form a point with a circle and a circle with an area, promote the formation of a new development pattern, accelerate the implementation of the relevant implementation opinions of Hainan Province's grid-tight medical and health service system, improve the medical consortium, hospital group, and other division of labor and cooperation models, and comprehensively improve diagnosis and treatment efficiency and resource utilization efficiency.

Third, accelerate the introduction and training of health professionals, expand the total number, and improve the quality. On the one hand, the establishment of first-class medical colleges, especially nursing colleges. Independently cultivate and stabilize health professionals. On the other hand, Hainan Province can rely on favorable policies to improve the salary of health professionals, innovate the post and staffing system, focus on introducing and training high-level health professionals, master the core medical technology, improve the level of medical technology, and create high-end medical services in the world. In addition, the policy should be appropriately tilted to the grass-roots level, accelerate the construction of the grass-roots health personnel team, innovate the assessment system of grass-roots health personnel, pay more attention to the improvement of their post-competence, service attitude, diagnosis and treatment level, and ensure the quality and quantity of grass-roots health personnel.

Fourth, strengthen international cooperation and exchanges to promote the sharing of medical and health resources. To maintain close contact with Beijing, Shanghai, Guangdong Province, and other places to strengthen technical exchanges and improve the level of medical technology and service efficiency. The introduction of domestic and foreign first-class medical institutions and research centers in Hainan to achieve co-construction, co-governance, and sharing to serve the construction of the Hainan Free Trade Port.

**Other modifications:**

We are very sorry. We examined the manuscript carefully and found that there were some sentences that needed to be changed and some places that had been added. These are highlighted in red in the manuscript. The details are as follows:

1. Line 20

**The modified：**Further description of co-contributing authors.

2. Lines 98-99

**Original text：**Hainan Province is the only free trade port in China

**The modified：**Hainan Province is currently the only free trade port with Chinese characteristics on the Chinese mainland,

3. Lines 140-141：Delete: Data envelopment analysis

**Original text：**Data envelopment analysis (DEA) is a non-parametric efficiency evaluation method first proposed by A. Charnes et al. in 1978.[9]

**The modified：**DEA is a non-parametric efficiency evaluation method first proposed by A. Charnes et al. in 1978.[13]

4. Lines 138, 206-207, 223, 235-236, 245-246

**The modified：**Bold font for the titles of the figures and tables

5. Lines 222：Delete Figure 1 and upload it separately.
6. Lines 301, 302, 305-306

Replace "Conclusion and policy implications" with "Conclusions and Suggestions" (Line 301)

Added content: The study found that (Line 302)

Added content: The, in Hainan Province (Lines 305-306)

7. Lines 308-312

**Original text：**In order to promote the high-quality development of the health industry of the Hainan Free Trade Port, the following suggestions are put forward.

**The modified：**Improving the efficiency of the allocation of medical and health resources is an important guarantee for promoting the development of the health industry of the Hainan Free Trade Port and an inevitable requirement for the high-quality construction of the Hainan Free Trade Port. In order to improve the efficiency of medical and health resources in Hainan Free Trade Port, the following suggestions are put forward.

8. As the "Introduction" section has been added, references have been added accordingly. (Lines 397-398, 403-412). The details are as follows: (With the revised manuscript, marked in yellow)

3. Chen Y. Research on equity and efficiency of health resource allocation in Yunnan Province [D]. Kunming University of Science and Technology,2021.

5. Lacko R, Hajduová Z, Bakalár T, Pavolová H. Efficiency and Productivity Differences in Healthcare Systems: The Case of the European Union. Int J Environ Res Public Health. 2022 Dec 22;20(1):178. doi: 10.3390/ijerph20010178. PMID: 36612499; PMCID: PMC9819540.

6. Xiang Yingru, WU Jin, WANG Cong, HUA Le, HUANG Xianhong. Evaluation of the allocation efficiency of provincial health resources before and after medical reform: Based on DEA and Tobit Methods [J]. Soft Sciences of Health, 2019,34(09):71-75.

7. Luo Liangqing, Hu Meiling. An Analysis on the Productive Efficiency of China's Regional Health and Care Services [J]. Journal of Statistics and Information,2008,No.89(02):47-51.

9. Modify the year and page number of a reference. (Lines 434-435)

**Original text：**10. Chiu Y, Chen Y. The analysis of Taiwanese bank efficiency: incorporating both external environment risk and internal risk. Econ Model. 2017; 26:456–63.

**The modified：**14. Chiu Y, Chen Y. The analysis of Taiwanese bank efficiency: incorporating both external environment risk and internal risk. Econ Model. 2009; 26:456–463.

Thank you very much for your attention and time. Looking forward to your reply.

Yours sincerely,

Yanhua GONG
