# Supplementary material for: Study on the allocation efficiency of medical and health resources in Hainan Province: Based on the super-efficiency SBM—Malmquist model
Source: PLoS One. 2024 Feb 29;19(2):e0294774. doi: 10.1371/journal.pone.0294774 (PMC10903814; doi:10.1371/journal.pone.0294774)
Supplement: Attachment — Submitted filename: Response to Reviewers.docx [file pone.0294774.s002.docx]

**Dear Editor and Reviewers:**

Thank you very much for your comments and professional advice on the manuscript (Title: Study on the allocation efficiency of medical and health resources in Hainan Province: Based on the super-efficiency SBM — Malmquist model. Number: PONE-D-23-10563R1). These opinions are helpful to improve the academic and rigorous nature of the manuscript. In the last round of revision, we carefully revised the manuscript and answered all the questions of the reviewers. Here we would like to thank the reviewers of the last round for their professional advice and recognition of our revised manuscript. Similarly, this time we are also holding a serious and steadfast learning attitude. According to your suggestions, we have carefully revised the manuscript and adjusted and polished some of the language. We hope the manuscript can be better. We have not changed much in the overall framework, but we changed the title to "Study on the allocation efficiency of medical and health resources in Hainan Province: Based on the super-efficiency SBM — Malmquist model" at your suggestion. In addition, the "Introduction" section and the "Discussion" section have been greatly revised. And the "Conclusions and suggestions" section has been deleted and reorganized. These are the focus of attention of experts. In a marked revision manuscript, we have highlighted your proposed revision in yellow, where the red font is to highlight that the author has added the editor's professional advice. Also, in this reply, we have highlighted some of the content in gray so that you can look more clear. Specific modification details are as follows:

**Dr. Agata Sielska (Editor) :**

Thank you very much for your professional advice on our first round of revisions. These suggestions help us to improve manuscript quality, make it more in line with journal requirements and attract readers' interest. We have added your suggestions to the body of the article. The following are our amendments and replies.

1. You have explained in the reply what you understand under "institutions" (referring to an input variable), but it is still not clear for a Reader who reads the manuscript only. Since "institutions" may also stand for the social structures (which is a popular concept in the economics), it is needed for you to disambiguate.

**The author's answer：**Thank you very much for your suggestion. We have added this to the article under Table 1 in the "Selection of indicators" section. (Lines 137-140) Specifically add as follows:

**Added content:** ^a^ Institutions mainly include hospitals, primary medical and health institutions, professional public health institutions, internet hospitals, and other medical and health institutions in the Hainan Provincial Health Statistics Yearbook. It reflects the overall size of the region's health systems. It is a public place to provide medical and health services and is one of the carriers.

2. It would be also very beneficial to include your answer to 5th comment from the Reviewer #1 in the main text ("What evidence or examples are provided to support the claim that economically developed areas with large populations have relatively higher allocation efficiency of health resources in Hainan Province?")

**The author's answer：**Thank you very much for your suggestion. We have already added this content to the "Discussion" section of the article at the appropriate place. At the same time, we also cite some studies by other scholars to illustrate this point. (Lines 298-320) Specifically add as follows:

**Added content:** The obvious differences among regions also reflect the unbalanced efficiency of medical resource allocation. The economically developed and populous region of Hainan Province has relatively high health resource allocation efficiency. Haikou City is the capital city of Hainan Province, and its annual gross domestic product (GDP) and population are the largest. Table 2 from the study also found that Haikou City has the highest overall efficiency. This may be related to the government's deepening reform of the healthcare system, adequate investment, and management. Economically developed regions usually have higher levels of investment in health resources, including medical facilities, equipment, and personnel, and such investment can provide better allocation and utilization of health resources and improve efficiency. They usually have more advanced medical technologies and management experience, which can help optimize health resource allocation and improve efficiency. For example, the application of 5G technology and electronic medical records can facilitate hierarchical treatment and improve the efficiency of healthcare organizations. The research and development of new drugs and improvements in surgical techniques can improve the efficiency of health resource allocation. Developed regions usually have better health policies and can receive greater policy concessions. For example, Haikou has many targeted health system integration and innovation measures. Many pilot measures will be implemented first in Haikou City. Areas with large populations usually have higher demand for health services, which prompts the government and healthcare organizations to increase investment in health. In addition, economically developed and populous areas are likely to be better educated and more aware of health care, which also drives the efficient use of health resources. This is consistent with Hang Ranfeng's study of health resource allocation efficiency in Jiangsu Province (2022) [18] and Huang Lili's study of health resource allocation efficiency in Hunan Province (2022) [19] .

3. It would be also very beneficial to include your answer to 2nd comment from Rewiever #2 in the main text ("It is incomprehensible to refer to a free port, what impact does it have on the functioning of health care? Are there any dependencies?"

**The author's answer：**Thank you very much for your suggestion. We have already added this content to the "Introduction" section of the article at the appropriate place. (Lines 91-101) Specifically add as follows:

**Added content:** The problem of unbalanced regional development has always existed in China. Hainan Province is currently the only free trade port with Chinese characteristics in mainland China, which is also known as the "test bed of national reform and opening up", which means that the successful experience of Hainan's reforms can be extended to other regions of China, and its construction is of great significance to China. [6] In the context of the free trade port, Hainan will have more development opportunities. At the same time, the efficiency of healthcare resource allocation, as an important indicator for safeguarding people's health, has also received widespread attention. Hainan's free trade port policy plays an important role in the operation of healthcare, for example, by providing more medical resources, promoting medical technology innovation, improving the quality of medical services, promoting the development of medical tourism, and optimizing the medical management system.

4. It would be also very beneficial to include your answer to 2nd comment from Rewiever #2 in the main text ("The authors focus on human resources, and is it not worth considering other resources, there is also a budget constraint that affects the efficiency of management, and thus also clinical effectiveness").

**The author's answer：**Thank you very much for your suggestion. We have already added this content to the "Discussion" section of the article at the appropriate place. (Lines 278-296) Specifically add as follows:

**Added content:** The reason for this is that, on the one hand, Hainan Province is unable to improve healthcare output through scientific and technological progress and innovation. On the other hand, the current investment in healthcare by the government of Hainan Province is too focused on hardware elements, and to a certain extent, it has neglected the improvement of healthcare technology, the quality of healthcare services, and the quality of health personnel. Of course, hardware inputs are essential, especially financial inputs. But these human resources inputs in health care are also very important. Medical and healthcare human resources are the core of the development of healthcare, which is a key factor. Human resources for health care have a significant impact on the development of health care in Hainan's free trade port and are important for the provision of high-quality health services, the promotion of medical tourism, and the protection of health security. Adequate human resources for health can provide a wider range and quality of health services and ensure that residents have timely access to medical and healthcare services. The development of a free trade port in Hainan implies the possibility of population mobility and growth, thus requiring sufficient human resources for health to meet the growing demand for health services. As a province with a highly developed tourism industry, Hainan has sufficient human resources for health to provide a high level of medical services and attract patients to the province. At the same time, Hainan's free trade port will require stronger human resources to ensure health safety and epidemic prevention and control.

5. I'd also suggest considering removal of the "free-port" from the title, as suggested by Reviewer #3, since you do not in fact discuss the free-port as a determinant, more as a background factor. However, following this advice is not a necessary condition for the acceptance of the paper - the current title summarizes the study well.

**The author's answer：**Thank you very much for your professional advice. After consultation and thinking, we also agree to delete the title "under the construction of free trade port" and change it to "Study on the allocation efficiency of medical and health resources in Hainan Province: Based on the super-efficiency SBM — Malmquist model". But the free trade port is still a background content of this study, in this background to explore a new development of Hainan Province. The free trade port policy has given Hainan a new opportunity for development. (Lines4-5)

**Original text：**Study on the allocation efficiency of medical and health resources in Hainan Province under the construction of Free Trade Port: Based on the super-efficiency SBM — Malmquist model

**The modified：**Study on the allocation efficiency of medical and health resources in Hainan Province: Based on the super-efficiency SBM — Malmquist model

6. Rewiever #3 suggests some further polishing of the language and I agree with them. Some sentences could be improved. Proof-reading by a naitve speaker may be helpful in this area.

**The author's answer：**Thank you very much for your suggestions. In order to improve our manuscript language, we consulted a specialized polishing agency before submitting the manuscript for the first time. After this revision, we discussed and revised the writing language seriously with a professional English tutor. All the suggestions he gave us were taken on board and adjusted accordingly in the manuscript. We apologize for causing you unnecessary trouble and hope that the revised version will meet your expectations.

(1) Lines 49-52

**Original text：**According to the "Healthy China 2030" Plan's Outline in 2016, by 2030, China's health institutions and systems will be improved, the development of the health sector will be more coordinated, healthy lifestyles will be popularized, and the quality and guarantee of health services will continue to improve.

**The modified：**The 2016 Outline of the "Healthy China 2030" Plan proposes that by 2030, China's health institution system will be more complete, the development of health undertakings will be more coordinated, healthy lifestyles will be popularized, and the quality of health services and the level of protection will be continuously improved. (Lines 49-52)

(2) Lines 244-246

**Original text：**However, through the super-efficiency SBM measurement, it is found that the comprehensive efficiency score of the samples is 0.975, indicating that the allocation efficiency of medical and health resources in Hainan Province is not well, which needs to be further improved.

**The modified：**However, through the super-efficiency SBM measurement, it is found that the comprehensive efficiency score of the samples is 0.975, which indicates that the efficiency of healthcare resource allocation in Hainan Province is not high and needs to be further improved.

(3) Line 248

**Original text：**The main reasons for the decline may have been these reasons.

**The modified：**The main reasons for the decline may be these:

(4) Lines 278-283

**Original text：**On the one hand, Hainan Province is not able to improve its medical and health output through scientific and technological progress and innovation. On the other hand, the current investment of the Hainan Provincial government in the medical and health field focuses too much on the hardware elements and neglects to some extent the improvement of medical and health technology, medical service quality, and health personnel quality.

**The modified：**The reason for this is that, on the one hand, Hainan Province is unable to improve healthcare output through scientific and technological progress and innovation. On the other hand, the current investment in healthcare by the government of Hainan Province is too focused on hardware elements, and to a certain extent, it has neglected the improvement of healthcare technology, the quality of healthcare services, and the quality of health personnel.

(5) Lines 341-346

**Original text：**The Hainan provincial government should further implement and optimize policies, accelerate the process of medical reform, improve detailed medical and health resource planning, and rationally allocate medical and health hardware and software resources according to population, geography, demand, and other factors in order to improve efficiency.

**The modified：**The Hainan government should further implement and optimize its policies, accelerate the process of healthcare reform, improve detailed healthcare resource planning, rationally allocate healthcare hardware and software resources based on demographic, geographic, and demand factors, and promote the expansion and balanced allocation of high-quality healthcare resources to improve efficiency.

**Reviewer #3:**

Thank you very much for your recognition of the manuscript and your professional suggestions. The following are our amendments and replies.

1. I do not suggest the authors to take the construction of free trade port in the title, because this point was not be discussed carefully. If possible, a DID or some other empirical estimation could be performed to estimate the policy effect, e.g. the construction of FTP.

**The author's answer：**Thank you very much for your professional advice. After consultation and thinking, we also agree to delete the title "under the construction of free trade port" and change it to "Study on the allocation efficiency of medical and health resources in Hainan Province: Based on the super-efficiency SBM — Malmquist model". But the free trade port is still a background content of this study, in this background to explore a new development of Hainan Province. The free trade port policy has given Hainan a new opportunity for development. (Lines4-5)

**Original text：**Study on the allocation efficiency of medical and health resources in Hainan Province under the construction of Free Trade Port: Based on the super-efficiency SBM — Malmquist model

**The modified：**Study on the allocation efficiency of medical and health resources in Hainan Province: Based on the super-efficiency SBM — Malmquist model

2. The main contribution and research questions was not reported carefully. The Introduction section should be rewritten and include a literature review to link the RQ, and the main contribution. "Foreign studies" should not be used.

**The author's answer：**Thank you very much for your professional advice. We have revised and improved the "Introduction" section. As it reflects the situation in China, we agree with your suggestion of not using some foreign studies. We have more prominently highlighted the major domestic studies and contributions in this area. We focus on reflecting the current reality in China as well as citing studies in other parts of China in this area, and we summarize the main contributions and shortcomings of the relevant literature in this area so as to better draw out the research questions of this paper. This study focuses on Hainan Province and is a study of how to improve the efficiency of healthcare resource allocation in Hainan Province. In the context of free trade port, this study adopts the super-efficient SBM model and Malmquist index to study the efficiency of healthcare resource allocation in Hainan Province from 2016 to 2020, analyze the status quo, problems, and reasons, and put forward rationalization suggestions to provide reference for the scientific formulation of healthcare development planning. (Lines 45-121)

**Original text：**The contradiction between the finiteness of health resources and the infinite demand of people is prominent.[1] How to make more effective use of the limited health resources, meet the needs of people at different levels of health services, optimize the distribution of health resources and boost the efficiency of use is a common problem faced by the global medical and health reform.[1, 2] According to the "Healthy China 2030" Plan's Outline in 2016, by 2030, China's health institutions and systems will be improved, the development of the health sector will be more coordinated, healthy lifestyles will be popularized, and the quality and guarantee of health services will continue to improve. The important premise of improving health levels and promoting health equity is the rational allocation and efficient utilization of medical and health resources. The improvement of the efficiency of the allocation of medical and health resources can not only promote the accessibility and quality of medical services but also play an important role in improving people's living standards and promoting economic growth. Since the implementation of China's new medical reform in 2009, the amount of health resource investment has generally increased, and great results have been achieved.

Foreign studies on the allocation of health resources started earlier, and the UK was the first country to conduct research on the allocation of health resources [3]. In 1976, the Resource Allocation Working Party (RAWP) was established in the United Kingdom to study the allocation of health resources. Research on the allocation of health resources in China began in the 1980s. With the development of the economy, the rapid increase in population, and the intensification of aging, people have put forward higher requirements for medical and health resources and services, and the government has continuously increased its investment in health resources. The number of health institutions, beds, and health technicians is increasing, and the service capacity of medical and health institutions is greatly improved, but the efficiency of medical and health resource allocation is still low, which may lead to increased investment in subsequent health resources but poor output. This not only fails to meet people's growing medical and health needs but also causes serious waste of medical and health resources, resulting in the rapid growth of medical costs, increasing people's medical burden, and restricting the sustainable and high-quality development of health and health undertakings, which is contrary to the ultimate goal of national health and health development.

How to make the best use of health resources is a hot topic for scholars at home and abroad. Data envelopment analysis ( DEA ) is the most mature and extensive method to study the efficiency of the allocation of medical and health resources. For example, Sayem Ahmed (2019) [4] used the DEA, Tobit model, and smooth bootstrap model to evaluate the technical efficiency of health systems in Asian countries and found that up to 91.3% of countries had inefficient utilization of health resources. Lacko R. (2022) [5] uses the DEA model and Malmquist index to compare the efficiency and productivity of EU countries. It is found that although countries joining the EU get a high technical efficiency value, the overall technical efficiency shows a downward trend, and the productivity of Western countries shows stagnation. Xiang Yingru (2020) [6] analyzed the efficiency of provincial health resources in China by using the BCC model of DEA and the Malmquist index. The study pointed out that after the implementation of medical reform in China, resources are still wasted, output is insufficient in some areas, and efficiency is low due to the excessive number of public hospitals and the serious aging of the population. Technological progress is a key factor affecting efficiency. Luo Liangqing (2008) [7] used the DEA model to study the production efficiency of medical and health services in the eastern, central, and western regions of China and found that the production efficiency of medical and health services in China was generally low. It can be seen from many related studies that the traditional DEA model has some limitations when studying the efficiency of the allocation of medical and health resources. Therefore, the method is improved in this article.

The problem of uneven development among different regions in China has always existed. Hainan Province is currently the only free trade port with Chinese characteristics on the Chinese mainland, and is also known as a "test bed for national reform and opening up ", which means that the successful experience of Hainan's reforms may be extended to other parts of China, and its construction is of great significance to China.[8] Hainan Province (108°37 '-- 111°03' east longitude, 18°10 '-- 20°10' north latitude) is located in the southernmost part of China, close to Southeast Asia, with a total land area of 35,400 square kilometers, and the number of permanent residents in 2020 is 10,123,400 people. In 2018, the Party Central Committee decided to support Hainan in gradually exploring and steadily deepening the construction of a free trade port with Chinese characteristics, which has helped the province comprehensively deepen reform. Hainan is committed to building Health Island and Longevity Island. Under the free trade port policy, Hainan has focused on institutional integration innovation and made major breakthroughs in health care. However, with the increase in population, the intensification of aging, and the change of disease spectrum, the current medical and health resources in Hainan Province are obviously in shortage, and there are outstanding problems such as imbalance, inequity, low efficiency, and lack of human resources. In order to effectively cope with future changes, Hainan must speed up the adjustment and optimization of medical and health resources and improve the efficiency of resource utilization, so as to provide an important guarantee for the high-quality development of health services in Hainan and escort the construction of free trade ports. At present, more attention is paid to the ecological environment and trade of Hainan Province at home and abroad, while there are few related types of research on health status, especially those on Hainan Province under the policy of free trade port with Chinese characteristics. In this study, the super-efficiency SBM model and Malmquist index were used to study the allocation efficiency of medical and health resources in Hainan Province during 2016-2020, analyze the current situation, problems, and causes, and put forward reasonable suggestions to provide a reference for scientific health development planning.

**The modified：**The contradiction between the limited nature of health resources and people's unlimited needs is prominent. [1] How to make more effective use of limited health resources, meet the needs of people at different levels for health services, optimize the allocation of health resources, and improve the efficiency of their use is a common problem facing global healthcare reform. [1,2] The 2016 Outline of the "Healthy China 2030" Plan proposes that by 2030, China's health institution system will be more complete, the development of health undertakings will be more coordinated, healthy lifestyles will be popularized, and the quality of health services and the level of protection will be continuously improved. An important prerequisite for improving health and promoting health equity is the rational allocation and effective utilization of medical and health resources. Improving the efficiency of healthcare resource allocation not only promotes the accessibility and quality of healthcare services but also plays an important role in raising people's living standards and promoting economic growth. Since the implementation of China's new healthcare reform in 2009, the investment in health resources has generally increased and achieved great results.

Research on health resource allocation in China began in the 1980s. With the development of the economy, the rapid increase in population, and the intensification of aging, people have put forward higher requirements for medical and health resources and services, and the government has continuously increased its investment in health resources. The number of health institutions, beds, and health technicians has continued to increase, and the service capacity of health institutions has greatly improved, but the efficiency of the allocation of health resources is still low, which may lead to subsequent increases in the input of health resources and poor output. This not only fails to meet the growing health care needs of the people but also results in a serious waste of health care resources, leading to a rapid increase in health care costs, aggravating the health care burden of the people, restricting the sustained and high-quality development of health care, and running counter to the ultimate goal of the country's health and health development. Ye Yizhong (2022) [19] used the three-stage SBM model and Malmquist index method to statically and dynamically evaluate the allocation efficiency of healthcare resources in 31 provinces (autonomous regions and municipalities directly under the central government, except for Hong Kong, Macao, and Taiwan) in China from 2015 to 2020. His study found that the allocation efficiency of health resources in China in 2015–2020 is low and on a downward trend, with obvious constraints on technological progress, large differences in allocation efficiency between regions and provinces, and significant impacts on the gross domestic product of each region, the number of permanent residents, and the level of urbanization. Xiang Yingru (2020) [6] analyzed the efficiency of health resources at the provincial level in China using the BCC model of DEA and the Malmquist index. The study pointed out that after the implementation of China's health care reform, due to the excessive number of public hospitals and the serious aging of the population, there are still problems such as waste of resources, insufficient output in some areas, and inefficiency. Technological progress is a key factor affecting efficiency. Luo Liangqing (2008) [7] utilized the DEA model to study the production efficiency of healthcare services in China's eastern, central, and western regions and found that China's healthcare service production efficiency is generally low. A large number of research results provide theoretical guidance for the research idea in this paper. From many related studies, it can be concluded that DEA is the most mature and widely used method to study the efficiency of healthcare resource allocation. However, the traditional DEA model has certain limitations in studying healthcare resource allocation efficiency. Therefore, this paper improves the method.

The problem of unbalanced regional development has always existed in China. Hainan Province is currently the only free trade port with Chinese characteristics in mainland China, which is also known as the "test bed of national reform and opening up", which means that the successful experience of Hainan's reforms can be extended to other regions of China, and its construction is of great significance to China. [6] In the context of the free trade port, Hainan will have more development opportunities. At the same time, the efficiency of healthcare resource allocation, as an important indicator for safeguarding people's health, has also received widespread attention. Hainan's free trade port policy plays an important role in the operation of healthcare, for example, by providing more medical resources, promoting medical technology innovation, improving the quality of medical services, promoting the development of medical tourism, and optimizing the medical management system. Hainan Province (108°37′–111°03′ E, 18°10′–20°10′ N) is located in the southernmost part of China, close to Southeast Asia, with a land area of 35,400 square kilometers and a resident population of 10,123,400 in 2020. In 2018, the Central Committee of the CPC decided to support Hainan to progressively explore and steadily deepen the construction of a free trade port with Chinese characteristics, helping Hainan to deepen its reforms comprehensively. Hainan is committed to building a healthy and long-lived island. Under the free trade port policy, Hainan focuses on system integration and innovation and has made significant breakthroughs in the field of healthcare. with the increase in population, the intensification of aging, and the change of disease spectrum, the current allocation of healthcare resources in Hainan Province is unreasonable, and the quality of healthcare services and the utilization of healthcare resources are inefficient, which seriously restricts the sustainable development of healthcare and wellness. Therefore, it is of great significance for Hainan Province to study how to improve the efficiency of healthcare resource allocation in Hainan Province to meet the people's demand for healthcare services, provide an important guarantee for the high-quality development of Hainan's healthcare industry, and escort the construction of the Free Trade Port. In the context of free trade port, this study adopts the super-efficient SBM model and Malmquist index to study the efficiency of healthcare resource allocation in Hainan Province from 2016 to 2020, analyze the status quo, problems, and reasons, and put forward rationalization suggestions to provide reference for the scientific formulation of healthcare development planning.

3. The methodology should be reported according to the marginal contribution of this manuscript, where "other indicators" is very clear. Maybe, the usage of per capita value is better.

**The author's answer：**Thank you very much for your suggestions. This paper has mentioned some of the marginal contributions that can be reacted to by this study in the “Discussion” section. For example, when analyzing the reasons for the decrease in comprehensive efficiency, the second reason is mentioned that Hainan Province has a corresponding increase in capital, equipment, and talent under the policy of free trade port, but the efficiency decreases instead of increasing. (Lines 258-267) When analyzing the results of dynamic efficiency, it is also found that technical efficiency, pure technical efficiency, and scale efficiency were all positive in 2016-2017, indicating that technical efficiency contributes to the increase of total factor productivity. The decline of total factor productivity in 2017 - 2020 is affected by both scale efficiency and technological progress, and it is more affected by technological setbacks. (Lines 273-277) When analyzing regional efficiency differences, this study also mentions that the management model should be reformed and medical services optimized to improve utilization efficiency, rather than compressing inputs, which also indicates that over time and with policy changes, these need to be innovated and injected with "fresh blood". (Lines 324-326) The application of marginal effects We focus on exploring the extent to which input influences have a marginal impact on the allocation efficiency of medical and health resources. (This is what we are currently working on.)

Using value per capita (which we call gross domestic product per capita) as an indicator is a very good suggestion. The "Other indicators" are our auxiliary indicators. We are thinking more of the gross domestic product per capita (GDP per capita) as our next study of impact factors. This we think will be more meaningful. The level of social and economic development directly affects government finance and residents' living standards, affects the accessibility of medical and health services, and further affects the supply and demand of medical and health resources. The important influence of the level of economic development on the allocation efficiency of healthcare resources can also be fully realized from the study of Wei Kezhen, Zhou Liting, and others.

[1] Wei Kezhen. Research on measurement and influencing factors of allocation efficiency of medical and health resources in China [D]. Shaanxi Normal University,2018.

[2] Zhou L T. Study on efficiency of medical and health resource allocation and its influencing factors in Jiangsu Province [D]. Harbin University of Commerce,2022.

**Article Related Content:**

Second, it may have something to do with the construction of free trade ports in Hainan Province starting in 2018. Hainan has given priority to institutional integration and innovation in its development. Various preferential policies have been formulated and introduced to attract capital, talent, high-end equipment, and other resources to Hainan, breaking the state of self-sufficiency in the past. With the deepening of the reform of the medical system, medical and health resources are being restructured and adjusted, and there is a phenomenon of underutilization and reasonable operation, resulting in a decline in efficiency instead of improvement. This also reflects from another side that no matter how much health resources are invested, if the resources are not reasonably and fully utilized, the efficiency of resource allocation will decline and the output will be poor. (Lines 258-267)

Malmquist index shows that technical efficiency, pure technical efficiency, and scale efficiency have positive growth in 2016-2017, indicating that technical efficiency contributes to the improvement of total factor productivity. The decline of total factor productivity from 2017 to 2020 is influenced by both scale efficiency and technological progress and is greatly influenced by technological regress. (Lines 273-277)

Therefore, it is necessary for each district and county government to reform the management mode, optimize the medical service and improve the service utilization efficiency, rather than compress the input. (Lines 324-326)

4. If possible, longer study period could contribute more.

**The author's answer：**We started this research work in August 2021. After repeated research, searching for information, consulting relevant experts, and continuous revision, we have enough data and evidence to do this research. During the period, we wrote the "High-quality Development Report of Hainan Health Industry in 2021" and "High-quality Development Report of Hainan Health Industry in 2022" for the Hainan Provincial government, both of which were highly evaluated by them and considered to be operable and practical. In addition, we have also published articles on the allocation of medical and health resources in Hainan Province in relevant journals at home and abroad. We are well aware of the importance of the allocation of medical and health resources, and we also know that perhaps our current research is not enough, and there are still places to study and study hard. We will do our best to make greater contributions in this area.

5. The discussion should be reorganized according to the main findings, contributions, and a detailed discussion for related literature and study for other provinces of China is needed.

**The author's answer：**Thank you very much for your suggestions. We have reorganized and revised the "Discussion" section and introduced relevant studies from other provinces in China. The "Discussion" section includes the following points: the comprehensive situation, dynamic situation and regional differences of the allocation efficiency of medical and health resources in Hainan Province. Studies from other Chinese regions, such as Chongqing, Jiangsu Province, and Hunan Province, have also been introduced. (Lines 242-326)

**Original text：**With the construction of the Hainan Free Trade Port, the demand for population and health services increases, and the allocation of health resources also increases year by year. However, through the super-efficiency SBM measurement, it is found that the comprehensive efficiency score of the samples is 0.975, indicating that the allocation efficiency of medical and health resources in Hainan Province is not well, which needs to be further improved. 2017 was relatively good, but the comprehensive efficiency from 2019 to 2020 was all less than 1, and there was a downward trend. The main reasons for the decline may have been these reasons. First, in 2017, it was emphasized in the Key Tasks of Deepening the Reform of the Medical and Health System in 2017 that the year 2017 was a key year for China to form a systematic framework of the basic medical and health system and complete the phased objectives of the medical reform. Therefore, China promulgated a number of medical reform policies, such as the cancellation of the drug price addition policy, the comprehensive launch of the medical consortium pilot work, and the implementation of a hierarchical diagnosis and treatment system. The medical and health service system in our country has broken the inherent mode under the effects of this series of medical reforms. During the initial period of the implementation of the new and old policies, it has exerted influence on the normal operation of health undertakings. Second, it may have something to do with the construction of free trade ports in Hainan Province starting in 2018. Hainan has given priority to institutional integration and innovation in its development. Various preferential policies have been formulated and introduced to attract capital, talent, high-end equipment, and other resources to Hainan, breaking the state of self-sufficiency in the past. With the deepening of the reform of the medical system, medical and health resources are being restructured and adjusted, and there is a phenomenon of underutilization and reasonable operation, resulting in a decline in efficiency instead of improvement. This also reflects from another side that no matter how much health resources are invested, if the resources are not reasonably and fully utilized, the efficiency of resource allocation will decline and the output will be poor. Third, due to the impact of the global COVID-19 epidemic in 2019–2020, health resources have been urgently deployed, including equipment, beds, personnel and other resources, focusing on the allocation of the most affected areas. More beds were transferred to treat COVID-19 patients. Medical resources are diverted to emergency response to the epidemic, thus affecting the original efficiency.

Malmquist index shows that technical efficiency, pure technical efficiency, and scale efficiency have positive growth in 2016-2017, indicating that technical efficiency contributes to the improvement of total factor productivity. The decline of total factor productivity from 2017 to 2020 is influenced by both scale efficiency and technological progress and is greatly influenced by technological regress. On the one hand, Hainan Province is not able to improve its medical and health output through scientific and technological progress and innovation. On the other hand, the current investment of the Hainan Provincial government in the medical and health field focuses too much on the hardware elements and neglects to some extent the improvement of medical and health technology, medical service quality, and health personnel quality.

The obvious differences among regions also reflect the unbalanced efficiency of medical resource allocation. From the perspective of comprehensive efficiency, Haikou, as the capital city of Hainan Province, has a relatively developed economy and a better allocation efficiency of health resources, which may be related to the government's deepening of the reform of the medical system, with sufficient input and in place management. However, from the perspective of total factor productivity, Haikou City is restricted by scale efficiency, indicating that the medical scale of Haikou City should be properly expanded. Inefficient cities and counties with large areas and small populations, there are redundant inputs and insufficient output, and it is difficult to make full use of health resources. Therefore, it is necessary for each district and county government to reform the management mode, optimize the medical service and improve the service utilization efficiency, rather than compress the input.

**The modified：**With the construction of the Hainan Free Trade Port, the demand for population and health services increases, and the allocation of health resources also increases year by year. However, through the super-efficiency SBM measurement, it is found that the comprehensive efficiency score of the samples is 0.975, which indicates that the efficiency of healthcare resource allocation in Hainan Province is not high and needs to be further improved. 2017 was relatively good, but the comprehensive efficiency from 2019 to 2020 was all less than 1, and there was a downward trend. The main reasons for the decline may be these: First, in 2017, it was emphasized in the Key Tasks of Deepening the Reform of the Medical and Health System in 2017 that the year 2017 was a key year for China to form a systematic framework of the basic medical and health system and complete the phased objectives of the medical reform. Therefore, China promulgated a number of medical reform policies, such as the cancellation of the drug price addition policy, the comprehensive launch of the medical consortium pilot work, and the implementation of a hierarchical diagnosis and treatment system. The medical and health service system in our country has broken the inherent mode under the effects of this series of medical reforms. During the initial period of the implementation of the new and old policies, it has exerted influence on the normal operation of health undertakings. Second, it may have something to do with the construction of free trade ports in Hainan Province starting in 2018. Hainan has given priority to institutional integration and innovation in its development. Various preferential policies have been formulated and introduced to attract capital, talent, high-end equipment, and other resources to Hainan, breaking the state of self-sufficiency in the past. With the deepening of the reform of the medical system, medical and health resources are being restructured and adjusted, and there is a phenomenon of underutilization and reasonable operation, resulting in a decline in efficiency instead of improvement. This also reflects from another side that no matter how much health resources are invested, if the resources are not reasonably and fully utilized, the efficiency of resource allocation will decline and the output will be poor. Third, due to the impact of the global COVID-19 epidemic in 2019–2020, health resources have been urgently deployed, including equipment, beds, personnel and other resources, focusing on the allocation of the most affected areas. More beds were transferred to treat COVID-19 patients. Medical resources are diverted to emergency response to the epidemic, thus affecting the original efficiency.

Malmquist index shows that technical efficiency, pure technical efficiency, and scale efficiency have positive growth in 2016-2017, indicating that technical efficiency contributes to the improvement of total factor productivity. The decline of total factor productivity from 2017 to 2020 is influenced by both scale efficiency and technological progress and is greatly influenced by technological regress. This is consistent with the studies of Cai Zihan (2022) [16] and Tian Haoguo (2022) [17]. The reason for this is that, on the one hand, Hainan Province is unable to improve healthcare output through scientific and technological progress and innovation. On the other hand, the current investment in healthcare by the government of Hainan Province is too focused on hardware elements, and to a certain extent, it has neglected the improvement of healthcare technology, the quality of healthcare services, and the quality of health personnel. Of course, hardware inputs are essential, especially financial inputs. But these human resources inputs in health care are also very important. Medical and healthcare human resources are the core of the development of healthcare, which is a key factor. Human resources for health care have a significant impact on the development of health care in Hainan's free trade port and are important for the provision of high-quality health services, the promotion of medical tourism, and the protection of health security. Adequate human resources for health can provide a wider range and quality of health services and ensure that residents have timely access to medical and healthcare services. The development of a free trade port in Hainan implies the possibility of population mobility and growth, thus requiring sufficient human resources for health to meet the growing demand for health services. As a province with a highly developed tourism industry, Hainan has sufficient human resources for health to provide a high level of medical services and attract patients to the province. At the same time, Hainan's free trade port will require stronger human resources to ensure health safety and epidemic prevention and control.

The obvious differences among regions also reflect the unbalanced efficiency of medical resource allocation. The economically developed and populous region of Hainan Province has relatively high health resource allocation efficiency. Haikou City is the capital city of Hainan Province, and its annual gross domestic product (GDP) and population are the largest. Table 2 from the study also found that Haikou City has the highest overall efficiency. This may be related to the government's deepening reform of the healthcare system, adequate investment, and management. Economically developed regions usually have higher levels of investment in health resources, including medical facilities, equipment, and personnel, and such investment can provide better allocation and utilization of health resources and improve efficiency. They usually have more advanced medical technologies and management experience, which can help optimize health resource allocation and improve efficiency. For example, the application of 5G technology and electronic medical records can facilitate hierarchical treatment and improve the efficiency of healthcare organizations. The research and development of new drugs and improvements in surgical techniques can improve the efficiency of health resource allocation. Developed regions usually have better health policies and can receive greater policy concessions. For example, Haikou has many targeted health system integration and innovation measures. Many pilot measures will be implemented first in Haikou City. Areas with large populations usually have higher demand for health services, which prompts the government and healthcare organizations to increase investment in health. In addition, economically developed and populous areas are likely to be better educated and more aware of health care, which also drives the efficient use of health resources. This is consistent with Hang Ranfeng's study of health resource allocation efficiency in Jiangsu Province (2022) [18] and Huang Lili's study of health resource allocation efficiency in Hunan Province (2022) [19] . However, from the perspective of total factor productivity, Haikou City is restricted by scale efficiency, indicating that the medical scale of Haikou City should be properly expanded. Inefficient cities and counties with large areas and small populations, there are redundant inputs and insufficient output, and it is difficult to make full use of health resources. Therefore, it is necessary for each district and county government to reform the management mode, optimize the medical service and improve the service utilization efficiency, rather than compress the input.

6. The last section is very lengthy and most of the suggestions are unnecessary without solid research findings. A more concise conclusion and policy implication section could bring more flowability.

**The author's answer：**After careful consideration and discussion, we agree with your views. We have made deletions and minor changes to the "suggestions" section to make the suggestions more flexible and operational. Specific modifications are as follows. (Lines 340-372)

**Original text：**First, Hainan should continue to take institutional integration and innovation as the "starting point" to improve the ability of modern governance of health services. The "14th Five-Year Plan for Hygiene and Health of Hainan Province" issued by Hainan Province in 2021 emphasizes that Hainan must accelerate the expansion and balanced distribution of high-quality medical and health resources and improve the allocation efficiency of medical and health resources. The Hainan provincial government should further implement and optimize policies, accelerate the process of medical reform, improve detailed medical and health resource planning, and rationally allocate medical and health hardware and software resources according to population, geography, demand, and other factors in order to improve efficiency. Accelerate the development of key health projects in Hainan Province. For example, promote the implementation of the global "One Health" demonstration project in Hainan Province through publicity and improvement of the medical and prevention integration governance model; Through the high-level equipment of hardware and software facilities, increase the degree of policy openness, pay attention to medical innovation and achievement transformation, and promote the process of the Boao Lecheng International Medical Tourism Pilot Zone; Based on the unique natural environment of Hainan Province, it is necessary to promote the rational development of the healthcare industry.

Second, Hainan should balance the layout and optimize the allocation of health resources, and speed up the process of promoting graded diagnosis and treatment and "Internet Plus" medical treatment. Strengthen supervision and management, appropriately expand the scale of medical services in big cities, and for inefficient cities and counties, and improve the efficiency of service utilization by means of technology introduction, graded diagnosis and treatment, and "Internet +" medical treatment while ensuring adequate input. Speed up the building of economic circles in Haikou, Sanya, and Dan Ocean, form a point with a circle and a circle with an area, promote the formation of a new development pattern, accelerate the implementation of the relevant implementation opinions of Hainan Province's grid-tight medical and health service system, improve the medical consortium, hospital group, and other division of labor and cooperation models, and comprehensively improve diagnosis and treatment efficiency and resource utilization efficiency.

Third, accelerate the introduction and training of health professionals, expand the total number, and improve the quality. On the one hand, the establishment of first-class medical colleges, especially nursing colleges. Independently cultivate and stabilize health professionals. On the other hand, Hainan Province can rely on favorable policies to improve the salary of health professionals, innovate the post and staffing system, focus on introducing and training high-level health professionals, master the core medical technology, improve the level of medical technology, and create high-end medical services in the world. In addition, the policy should be appropriately tilted to the grass-roots level, accelerate the construction of the grass-roots health personnel team, innovate the assessment system of grass-roots health personnel, pay more attention to the improvement of their post-competence, service attitude, diagnosis and treatment level, and ensure the quality and quantity of grass-roots health personnel.

Fourth, strengthen international cooperation and exchanges to promote the sharing of medical and health resources. To maintain close contact with Beijing, Shanghai, Guangdong Province, and other places to strengthen technical exchanges and improve the level of medical technology and service efficiency. The introduction of domestic and foreign first-class medical institutions and research centers in Hainan to achieve co-construction, co-governance, and sharing to serve the construction of the Hainan Free Trade Port.

**The modified：**First, Hainan should continue to take institutional integration and innovation as the "starting point" to improve the ability of modern governance of health services. The Hainan government should further implement and optimize its policies, accelerate the process of healthcare reform, improve detailed healthcare resource planning, rationally allocate healthcare hardware and software resources based on demographic, geographic, and demand factors, and promote the expansion and balanced allocation of high-quality healthcare resources to improve efficiency. Accelerate the development of key health projects in Hainan Province.

Second, Hainan should balance the layout and optimize the allocation of health resources, and speed up the process of promoting graded diagnosis and treatment and "Internet Plus" medical treatment. Strengthen supervision and management, appropriately expand the scale of medical services in big cities, and for inefficient cities and counties, and improve the efficiency of service utilization by means of technology introduction, graded diagnosis and treatment, and "Internet +" medical treatment while ensuring adequate input. Accelerate the implementation of the relevant implementation opinions of Hainan Province's grid-tight medical and health service system, improve the medical consortium, hospital group, and other division of labor and cooperation models, and comprehensively improve diagnosis and treatment efficiency and resource utilization efficiency.

Third, accelerate the introduction and training of health professionals, expand the total number, and improve the quality. On the one hand, the establishment of first-class medical colleges, especially nursing colleges. Independently cultivate and stabilize health professionals. On the other hand, Hainan Province can rely on favorable policies to improve the salary of health professionals, innovate the post and staffing system, focus on introducing and training high-level health professionals, master the core medical technology, improve the level of medical technology, and create high-end medical services in the world. In addition, the policy should be appropriately tilted to the grass-roots level and accelerate the construction of the grass-roots health personnel team.

Fourth, strengthen international cooperation and exchanges to promote the sharing of medical and health resources. To maintain close contact with Beijing, Shanghai, Guangdong Province, and other places to strengthen technical exchanges and improve the level of medical technology and service efficiency. The introduction of domestic and foreign first-class medical institutions and research centers in Hainan to achieve co-construction, co-governance, and sharing to serve the construction of the Hainan Free Trade Port.

7. The language level should be improved, and the authors should report the work to international readers with its marginal contribution.

**The author's answer：**Thank you very much for your suggestions. In order to improve our manuscript language, we consulted a specialized polishing agency before submitting the manuscript for the first time. After this revision, we discussed and revised the writing language seriously with a professional English tutor. All the suggestions he gave us were taken on board and adjusted accordingly in the manuscript. We apologize for causing you unnecessary trouble and hope that the revised version will meet your expectations.

(1) Lines 49-52

**Original text：**According to the "Healthy China 2030" Plan's Outline in 2016, by 2030, China's health institutions and systems will be improved, the development of the health sector will be more coordinated, healthy lifestyles will be popularized, and the quality and guarantee of health services will continue to improve.

**The modified：**The 2016 Outline of the "Healthy China 2030" Plan proposes that by 2030, China's health institution system will be more complete, the development of health undertakings will be more coordinated, healthy lifestyles will be popularized, and the quality of health services and the level of protection will be continuously improved. (Lines 49-52)

(2) Lines 244-246

**Original text：**However, through the super-efficiency SBM measurement, it is found that the comprehensive efficiency score of the samples is 0.975, indicating that the allocation efficiency of medical and health resources in Hainan Province is not well, which needs to be further improved.

**The modified：**However, through the super-efficiency SBM measurement, it is found that the comprehensive efficiency score of the samples is 0.975, which indicates that the efficiency of healthcare resource allocation in Hainan Province is not high and needs to be further improved.

(3) Line 248

**Original text：**The main reasons for the decline may have been these reasons.

**The modified：**The main reasons for the decline may be these:

(4) Lines 278-283

**Original text：**On the one hand, Hainan Province is not able to improve its medical and health output through scientific and technological progress and innovation. On the other hand, the current investment of the Hainan Provincial government in the medical and health field focuses too much on the hardware elements and neglects to some extent the improvement of medical and health technology, medical service quality, and health personnel quality.

**The modified：**The reason for this is that, on the one hand, Hainan Province is unable to improve healthcare output through scientific and technological progress and innovation. On the other hand, the current investment in healthcare by the government of Hainan Province is too focused on hardware elements, and to a certain extent, it has neglected the improvement of healthcare technology, the quality of healthcare services, and the quality of health personnel.

(5) Lines 341-346

**Original text：**The Hainan provincial government should further implement and optimize policies, accelerate the process of medical reform, improve detailed medical and health resource planning, and rationally allocate medical and health hardware and software resources according to population, geography, demand, and other factors in order to improve efficiency.

**The modified：**The Hainan government should further implement and optimize its policies, accelerate the process of healthcare reform, improve detailed healthcare resource planning, rationally allocate healthcare hardware and software resources based on demographic, geographic, and demand factors, and promote the expansion and balanced allocation of high-quality healthcare resources to improve efficiency.

Thank you very much for your attention and time. Looking forward to your reply.

Yours sincerely,

Yanhua GONG
