# Supplementary material for: Study on the allocation efficiency of medical and health resources in Hainan Province: Based on the super-efficiency SBM—Malmquist model
Source: PLoS One. 2024 Feb 29;19(2):e0294774. doi: 10.1371/journal.pone.0294774 (PMC10903814; doi:10.1371/journal.pone.0294774)
Supplement: Attachment — Submitted filename: Response to Reviewers.docx [file pone.0294774.s003.docx]

**Dear Editor and Reviewers:**

Thank you very much for your comments and professional advice on the manuscript (Title: Study on the allocation efficiency of medical and health resources in Hainan Province: Based on the super-efficiency SBM — Malmquist model. Number: PONE-D-23-10563R3). These opinions are helpful to improve the academic and rigorous nature of the manuscript. In the previous review, we carefully revised the manuscript and answered all the questions raised by the reviewers. We would like to thank all reviewers for their professional advice and recognition of our revised manuscript. Similarly, this time we are also holding a serious and steadfast learning attitude. According to your suggestions, we have carefully revised the manuscript. We hope the manuscript can be better. We have not changed the overall framework of the manuscript, but the "suggestions" section has been modified considerably. In a marked revision manuscript, we have highlighted your proposed revision in yellow. Also, in this reply, we have highlighted some of the content in gray so that you can look more clear. Specific modification details are as follows:

**Reviewer #3:**

Thank you very much for your recognition of the manuscript and your professional suggestions. The following are our amendments and replies.

1. I strongly suggest the authors to reveal the reason to select research data span beginning form 2016. If longer data span is available, it should be incorparated into the work. If no available data is there, pls tell the readers in the manuscript.

**The author's answer：**Thank you very much for your professional advice. We add this content to the "Source of data" section. Based on the policy background, data availability, and trend changes, this paper selects the time span from 2016 to 2020 to study the allocation efficiency of medical and health resources in Hainan Province. However, the study on the allocation efficiency of medical and health resources in Hainan Province is a continuous study, and it is necessary to constantly supplement the complete relevant data to improve the allocation efficiency to the greatest extent with the development of time. Therefore, we will continue to pay attention to its development. (Lines 130-148) Specific additions are as follows:

**Added content:** This paper combines policy background, data availability, and trend changes to choose the time span of 2016–2020 to study the healthcare resource allocation efficiency in Hainan Province. In terms of policy background, since 2016, the Chinese government has proposed a series of policy measures to promote the efficiency of healthcare resource allocation, and the implementation of these policies has had a positive impact on the efficiency of healthcare resource allocation in Hainan Province. In addition, in 2018, under the policy of the Free Trade Port, Hainan Province is fully committed to deepening reform. Therefore, the choice of 2016 as the starting year has a certain policy representativeness, and the choice of 2020 as the cut-off year is because when this study was done, the data could only be obtained up to 2020. In terms of data availability, after 2016, the data related to the allocation of healthcare resources in Hainan Province were gradually improved, which can reflect the actual situation of healthcare resource allocation in Hainan Province in a more comprehensive way, and there were more missing data before 2016. Therefore, we chose the time span of 2016–2020 to ensure the accuracy and reliability of the data as much as possible. In terms of trend change, with the passage of time, the trend of healthcare resource allocation efficiency in Hainan Province will also change, especially in the context of the free trade port. By studying the time span of 2016–2020, the changing trend of healthcare resource allocation efficiency in Hainan Province before and after the implementation of the Free Trade Port can be more clearly grasped, thus providing a reference for policy formulation in Hainan Province.

2. The limitation on the methodology used in this paper should be bfiefly discussed, e.g. the spatial distribution of allocative efficeincy?

**The author's answer：**Thank you very much for your professional advice. This led us to think more deeply. We have supplemented the limitations of super-efficiency SBM in the "Super-efficiency SBM" section. (Lines 194-199) The limitations of the Malmquist index are supplemented in the "Malmquist index" section. (Lines 215-219) The details are as follows:

**Added content:**

However, the super-efficiency SBM model also has its limitations. First, this assessment method ignores some non-economic factors, such as environmental efficiency and social efficiency. Therefore, it does not fully assess the comprehensive efficiency. Secondly, it has high requirements for input and output data, but there are also data that are more difficult to obtain in practice, which may affect the assessment results. Third, it cannot reflect the spatial distribution of efficiency. (Lines 194-199)

The Malmquist index assesses technical efficiency change by calculating the product of technical progress and efficiency change. However, the biggest limitation of the method is that it ignores the source of technical progress. In other words, it is not known whether technical progress is driven by internal innovation or external introduction. This problem may lead to errors in the assessment results. (Lines 215-219)

3. The present policy suggestions are very long and of low correlation with the findings of this manuscript. Without the authors' calculation, major suggestions could also be "CORRECT". I suggest the authors to delete all the suggestions if they could not be directly derived from the Super-DEA models estimation result.

**The author's answer：**Thank you very much for your professional advice. We have made changes to the "suggestions" section. After careful consideration, we did not delete all the recommendations but kept the concise ones in light of our findings. According to our conclusions, there are obvious regional differences in the efficiency of healthcare resource allocation in Hainan Province. In addition, efficiency is affected by the dual factors of scale efficiency and technological progress, of which technological progress is the key factor. Therefore, we believe that the allocation efficiency can be improved by promoting the expansion and balanced allocation of high-quality medical resources, expanding the total number of personnel, improving the quality of personnel, and upgrading the level of technology. (Lines 365-376) Specific modifications are set out below:

**Original text：**Improving the efficiency of the allocation of medical and health resources is an important guarantee for promoting the development of the health industry of the Hainan Free Trade Port and an inevitable requirement for the high-quality construction of the Hainan Free Trade Port. In order to improve the efficiency of medical and health resources in Hainan Free Trade Port, the following suggestions are put forward.

First, Hainan should continue to take institutional integration and innovation as the "starting point" to improve the ability of modern governance of health services. The Hainan government should further implement and optimize its policies, accelerate the process of healthcare reform, improve detailed healthcare resource planning, rationally allocate healthcare hardware and software resources based on demographic, geographic, and demand factors, and promote the expansion and balanced allocation of high-quality healthcare resources to improve efficiency. Accelerate the development of key health projects in Hainan Province.

Second, Hainan should balance the layout and optimize the allocation of health resources, and speed up the process of promoting graded diagnosis and treatment and "Internet Plus" medical treatment. Strengthen supervision and management, appropriately expand the scale of medical services in big cities, and for inefficient cities and counties, and improve the efficiency of service utilization by means of technology introduction, graded diagnosis and treatment, and "Internet +" medical treatment while ensuring adequate input. Accelerate the implementation of the relevant implementation opinions of Hainan Province's grid-tight medical and health service system, improve the medical consortium, hospital group, and other division of labor and cooperation models, and comprehensively improve diagnosis and treatment efficiency and resource utilization efficiency.

Third, accelerate the introduction and training of health professionals, expand the total number, and improve the quality. On the one hand, the establishment of first-class medical colleges, especially nursing colleges. Independently cultivate and stabilize health professionals. On the other hand, Hainan Province can rely on favorable policies to improve the salary of health professionals, innovate the post and staffing system, focus on introducing and training high-level health professionals, master the core medical technology, improve the level of medical technology, and create high-end medical services in the world. In addition, the policy should be appropriately tilted to the grass-roots level and accelerate the construction of the grass-roots health personnel team.

Fourth, strengthen international cooperation and exchanges to promote the sharing of medical and health resources. To maintain close contact with Beijing, Shanghai, Guangdong Province, and other places to strengthen technical exchanges and improve the level of medical technology and service efficiency. The introduction of domestic and foreign first-class medical institutions and research centers in Hainan to achieve co-construction, co-governance, and sharing to serve the construction of the Hainan Free Trade Port.

**The modified：**Improving the efficiency of the allocation of medical and health resources is an important guarantee for promoting the development of the health industry of the Hainan Free Trade Port and an inevitable requirement for the high-quality construction of the Hainan Free Trade Port. According to the conclusions of the paper, in order to improve the efficiency of medical and health resources in Hainan Free Trade Port, Hainan Province should rationalize the allocation of medical hardware and software resources based on demographic, geographic, and demand factors, accelerate the promotion of hierarchical diagnosis and treatment and "Internet+" medical care, and promote the expansion and balanced allocation of high-quality medical resources to improve efficiency. In addition, Hainan Province should accelerate the introduction and cultivation of health professionals, focusing on the improvement of medical and health technologies, the quality of medical and health services, and the quality of medical and health personnel.

Thank you very much for your attention and time. Looking forward to your reply.

Yours sincerely,

Yanhua GONG
